# A process evaluation of a worksite vitality intervention among ageing hospital workers

**DOI:** 10.1186/1479-5868-8-58

**Published:** 2011-06-10

**Authors:** Jorien E Strijk, Karin I Proper, Allard J van der Beek, Willem van Mechelen

**Affiliations:** 1 Department of Public and Occupational Health, EMGO+ Institute for Health and Care Research, VU University Medical Center, van der Boechorststraat 7, 1081 BT Amsterdam, The Netherlands; 2 Body@Work, Research Center Physical Activity, Work and Health, TNO-VUmc, Amsterdam, The Netherlands

**Keywords:** process evaluation, ageing workers, vitality, lifestyle intervention

## Abstract

**Background:**

The process evaluation of the Vital@Work intervention was primary aimed at gaining insight into the context, dose delivered, fidelity, reach, dose received, and participants' attitude. Further, the differences between intervention locations were evaluated.

**Methods:**

Eligible for this study were 730 workers, aged ≥ 45 years, from two academic hospitals. Workers randomised to the intervention group (n = 367) received a 6-months intervention consisting a Vitality Exercise Programme (VEP) combined with three visits to a Personal Vitality Coach (PVC), aimed at goal setting, feedback, and problem solving. The VEP consisted of a guided yoga session, a guided workout session, and aerobic exercising without direct face-to-face instruction, all once a week. Data were collected by means of a questionnaire after the intervention, attendance registration forms (i.e. attendance at guided VEP group sessions), and coaching registration forms (filled in by the PVCs).

**Results:**

The dose delivered of the yoga and workout sessions were 72.3% and 96.3%. All PVC visits (100%) were offered. The reach for the yoga sessions, workout sessions and PVC visits was 70.6%, 63.8%, and 89.6%, respectively. When taken these three intervention components together, the reach was 52%. This differed between the two locations (59.2% versus 36.8%). The dose received was for the yoga 10.4 sessions/24 weeks and for the workout 11.1 sessions/24 weeks. The attendance rate, defined as the mean percentage of attended group sessions in relation to the total provided group sessions, for the yoga and workout sessions was 51.7% and 44.8%, respectively. For the yoga sessions this rate was different between the two locations (63.2% versus 46.5%). No differences were found between the locations regarding the workout sessions and PVC visits. Workers attended on average 2.7 PVC visits. Overall, workers were satisfied with the intervention components: 7.5 for yoga sessions, 7.8 for workout sessions, and 6.9 for PVC visits.

**Conclusions:**

The implementation of the intervention was accomplished as planned with respect to the dose delivered. Based on the reach, most workers were willing to attend the guided group sessions and the PVC visits, although there were differences between the locations and between intervention components. Overall, workers were positive about the intervention.

**Trial registration:**

Trial registration NTR1240

## Background

Because the workforce is rapidly ageing in the upcoming decades, there is an urgent need for workers who are able to prolong their working life in good health. Despite lack of sound documentation, it is assumed that vitality is closely related to health [1,2]. Vitality is related to both mental and physical factors of health [3-8]. Regarding the mental factors, vitality reflects well-being, lower levels of fatigue, higher levels of emotional energy, mental resilience, and perseverance [3-7]. With respect to the physical factors, vitality is characterised by high energy levels and feeling "strong and fit" [7]. Physical activity may improve both older workers' mental and physical components of vitality by favourably affecting mental health, well-being, and feelings of fatigue [9-12], as well as symptoms of physical illness, disability, immunological dysfunction [4] and through improved levels of health-related fitness, such as aerobic fitness (i.e. VO_2max_) (11).

As healthy lifestyle choices contribute to better health outcomes [9,13-16], an intervention aimed at an improved lifestyle is considered a potentially effective tool to keep older workers vital, promote their health, and thereby prolong labour participation of these older workers [1]. In intervention studies, to assess whether the intervention was successful or not, the emphasis is mostly placed on the effects of the intervention [17]. As a consequence, it remains unclear which intervention components cause the eventual positive effects (black-box principle) [18]. Lately, researchers of intervention studies realise more often that for better explanations of their study findings, a process evaluation is a useful approach [19]. This is because a process evaluation gives insight into what extent and how the intervention components are being derived by the provider, and to what extent the components are being received and used by the intervention recipient [20]. This information is useful to determine the degree to which the intervention was implemented and used as planned. This makes it possible for researchers to understand the relationship between specific program elements and intervention outcomes [17,19]. Also, a process evaluation gives information about inhibiting and facilitating factors of the intervention, which is useful to improve the development and implementation of future interventions.

In the Vital@Work study, a lifestyle intervention was developed to improve older workers' vitality and will be subsequently evaluated for effectiveness [1]. The intervention consisted of: 1) the Vitality Exercise Programme (VEP) combined with 2) three visits to a Personal Vitality Coach (PVC) which were aimed at goal setting, feedback, and problem solving. The VEP consisted of a guided yoga session, a guided workout session, and aerobic exercising without direct face-to-face instruction, all once a week. Supplementary, free fruit was provided at the guided group sessions of the VEP. The purpose of the study presented in this paper was to evaluate the process of the Vital@Work intervention by gaining isight into the context, dose delivered, fidelity, reach, dose received, and participants' attitude. Supplementary, the eventual differences between intervention locations were evaluated.

## Methods

### Study population

This process evaluation was part of the Vital@Work study, a Randomised Controlled Trial (RCT) evaluating a lifestyle intervention to promote older workers' vitality [1]. A total of 730 older workers (aged 45 years and over) were included in the Vital@Work study. Inclusion criteria were: 1) working at least 16 hours a week at the academic hospital, 2) written informed consent, and 3) no risk for developing adverse health effects when becoming physically active. This risk for adverse health effects was assessed by using the Physical Activity Readiness Questionnaire (PAR-Q) [21]. Workers were randomised to an intervention group (n = 367) or a control group (n = 363). Workers in the intervention group received the six months lasting Vital@Work intervention. At the start of the project, both workers in the intervention and the control group received once written information about a healthy lifestyle (i.e. diet, physical activity and relaxation) at the start of the project. The study protocol was approved by the Medical Ethics Committee of the VU University Center Amsterdam (VUmc) and of the Leiden University Medical Center (LUMC).

### The Vital@Work intervention

The Vital@Work intervention was evaluated at two academic hospitals in the Netherlands; VU University Medical Center Amsterdam (VUmc) and Leiden University Medical Center (LUMC). The intervention lasted 6 months and consisted of: 1) the Vitality Exercise Programme (VEP) combined with 2) three visits to a Personal Vitality Coach (PVC). Supplementary, free fruit was provided at the guided group sessions of the VEP.

#### The Vitality Exercise Programme (VEP)

The VEP consisted of: 1) a yoga group session once a week consisting of relaxation exercises, 2) a workout group session once a week consisting of aerobic and resistance exercises, and 3) aerobic exercises without direct face-to-face instruction. Both the yoga and workout sessions were guided by qualified yoga and fitness instructors, respectively. The guided group sessions were provided (in total 24 sessions during the intervention period of 6 months) in small groups (max. 16 persons), and lasted 45 minutes. It was prescribed that both the guided yoga and workout group sessions were provided in two time blocks on all working days (Monday till Friday): 1) during lunchtime, and 2) after working hours (i.e. after 4 pm). During the intervention period, to facilitate a healthy lifestyle, free fruit was provided at the guided yoga and workout group sessions. As for the aerobic exercises without direct face-to-face instruction, workers were prescribed by the PVC during the visits to perform once a week for at least 45 minutes vigorous physical activity without face-to-face instructions (e.g. fitness, running). To achieve improvement in aerobic fitness, workers were asked to exercise at an intensity comparable to the guided workout sessions. As an illustration of this intensity, workers got the instruction to exercise with an intensity at which they experience sweating and an increased respiration and heart beat.

#### Personal Vitality Coach (PVC) Visits

The first visit with the PVC was at the start of the intervention. The two follow-up visits were at 4-6 weeks and 10-12 weeks after the first PVC visit. The PVC visits, lasting 30 minutes each, were aimed at five items: 1) setting personal goals (i.e. losing weight; increasing aerobic fitness) and explanation of the goals of the VEP (a yoga session once a week; a workout session once a week; and aerobic exercise without direct face-to-face instruction once a week), 2) getting confidence in achieving formulated goals, 3) giving feedback on formulated goals, 4) discussing barriers for formulated goals, and 5) problem solving. At the first visit the items goal setting and getting confidence in achieving formulated goals were discussed. At the second and third visit, which were comparable content wise, the other three items were discussed. During a 4-hour training session, the PVC protocol and accompanying materials, such as the coaching registration forms, were explained to the six coaches. At location Amsterdam, the PVC visits were provided by three coaches; two human movement scientists and one health scientist. One coach did not finish the intervention because of a change of job. At location Leiden, the PVC visits were provided by three physical therapists. Although the coaches were not actively involved in the yoga and workout sessions, all coaches had experience with sport exercise training.

### Data collection

This process evaluation was based on the process elements as described by Steckler and Linnan [20] and included: 1) the context of the intervention (context), 2) the extent to which the activities of the intervention were executed as planned (dose delivered, fidelity), 3) the extent to which the workers were exposed to the intervention (reach, dose received), and 4) the workers' attitude towards the intervention (participants' attitude). These process variables are described in Additional file 1 Table 1. Except for the context of the intervention, data was collected using: 1) attendance registration forms of the guided yoga en workout sessions, 2) coaching registration forms, 3) a questionnaire after the intervention, and 4) a physical activity log. The attendance registration forms were used to asses the dose delivered, dose received, and fidelity of the group sessions. They were filled in by the fitness and yoga instructors at the start of each session. If sessions were cancelled (e.g. no availability of a yoga facility, absence of instructor, etc.), the reason, date and time of the cancelled session were registered by the instructor. There was an attendance registration form for each arranged guided yoga and workout session. The coaching registration forms were provided for each PVC visit and were used to assess the dose delivered, dose received, and fidelity of the PVC visits. The coaching registration forms were filled in by the PVC together with the worker, and described information as to date of the visit and the items to be discussed, which were indicated on the form. Information form the questionnaire was used to assess workers' attitude towards the intervention. The purpose of the physical activity log was to assess the dose received and fidelity of the once a week aerobic exercise session without face-to face instruction and this should have been registered by the worker during the first 12 weeks of the intervention (i.e. simultaneous to the PVC visits). Because workers had considerable problems keeping the log up-to-date and information was only gathered during the first 12 weeks of the intervention, the dose received and fidelity of the once a week aerobic exercise sessions without face-to-face instruction were not described in this paper.

#### Context of the intervention

The context consisted of a description of organisational and environmental factors concerning the Vital@Work intervention. As for the organisational factors, it was described whether: 1) there was management support for the implementation and evaluation of the Vital@Work intervention at the two participating hospitals, and 2) workers were allowed to participate during paid work hours. As for the environmental factors, the two intervention locations were described (i.e. distance to facilities).

#### Implementation of the intervention as planned

To gain insight into whether the intervention components were implemented as planned, the dose delivered of the guided group sessions and PVC sessions was measured and information was obtained as to the fidelity of the intervention.

##### Dose delivered

The dose delivered reflected the number of guided group sessions and PVC visits delivered by the providers. The dose delivered components measured were the guided yoga group sessions, the guided workout group sessions, and the PVC visits provided. The number of provided guided group sessions was measured using attendance registration forms. The numbers of provided PVC visits were measured by the PVCs using the coaching registration forms. The dose delivered rate (%) for the group sessions was defined as: the number of actual provided group sessions divided by the agreed number of group sessions. For the PVC visits the dose delivered rate was defined as: the number of actual provided PVC visits divided by the agreed number of PVC visits.

##### Fidelity

The fidelity of the intervention referred to the extent to which the Vital@Work intervention was implemented as planned. For this process element, the following items were measured:

• Whether the guided yoga and workout sessions provided were offered in accordance with the preliminary appointed time schedules

• Average group sizes of the provided yoga and workout group sessions

• Mean number of items discussed during the PVC visits

The attendance to the preliminary appointed time schedules for the group sessions and the average group sizes were measured using attendance registration forms. The group sizes of the guided group sessions of the VEP were calculated by summing the number of attending workers per guided group session. To assess the discussed items, information obtained from the coaching registration forms was used.

#### Workers' exposure to the intervention

The process elements 'reach' and 'dose received' were determined to identify the workers' exposure to the Vital@Work intervention.

##### Reach

The reach of the Vital@Work intervention indicated the proportion of the older workers that participated in the intervention. In order to determine the reach, the percentage of workers that had participated at least once in each intervention component (i.e. ≥ 1 PVC visit, and ≥ 1 yoga session, and ≥ 1 workout session) was measured. The attendance to the VEP guided sessions and the PVC visits were measured using: 1) the attendance registrations forms for the guided group sessions, and 2) the coaching registration forms, respectively.

##### Dose received

The dose received referred to the extent to which the older workers were engaged to the intervention. The following items for the dose received were measured:

• Mean number and mean attendance rate (%) of the guided group sessions

• Mean number of attended PVC visits

The attended guided group sessions and PVC visits were measured using attendance registration forms and coaching registration form, respectively. The attendance rate (%) was defined as the mean percentage of attended guided group sessions in relation to the total provided group sessions.

#### Workers' attitude towards the intervention

The workers' attitude referred to their overall opinion and satisfaction towards the Vital@Work intervention. To assess workers' opinion, they were asked to rate their opinion about the guided yoga and workout sessions of the VEP and the PVC visits, on a scale from 0 to 10 (very bad [0] to excellent [10]). Preceding the start of the intervention, workers had indicated during focus group interviews held to develop the intervention [1] that guidance about how to perform exercises without getting injured during the group sessions involving physical activity was an important facilitator for participation. Therefore, workers were also asked to rate their opinion with regard to the training guidance of the workout and yoga instructors on a 5-point scale (excellent [1] to very poor [5] guidance).

### Statistical analysis

In cases where the variables were displayed as mean values, statistical differences between the two location were tested. This was done by an independent t-test for continuous variable was and by a Chi-square test in case of a dichotomous variable. For all analysis, SPSS version 15.0 was used. Statistical significance was defined as p < 0.05.

## Results

### Context of the intervention

As for the organisational factors, at both participating hospitals the implementation of the Vital@Work intervention was approved by the upper management (i.e. board of directors, the work counsels' committee, HR management, and the occupational health department). At location Amsterdam, a written communication was sent to all supervisors and team leaders by email to document that support. This was not done at location Leiden since the upper management did prefer to be not included into any practical affairs. However, at location Leiden the supervisors and team leaders of the participating departments, were as often as possible personally informed by the director of the occupational health department. In Amsterdam, the Vital@Work intervention was part of the integral health policy of the hospital and seen as a pilot for future health promotion policy. In Leiden, the Vital@Work study was an independent project. At both locations, workers had to participate to the intervention outside working hours.

As for the environmental factors, the Vital@Work intervention was provided at two academic hospitals in the Netherlands; VU medical centre Amsterdam and Leids University medical centre Leiden. At location Amsterdam, the intervention was provided by the VU university sport centre, which facilities are mainly situated at the university campus. As a consequent, it was possible to provide the guided workout and yoga sessions within a distance of less than 10 minutes walking from the worksite. The workout sessions were given by instructors at the sport centre on the campus, the yoga sessions were given yoga instructors in a physical therapy treatment room within the hospital itself. At location Leiden, the intervention was provided by an independent physical therapy practice, which had also sport exercise facilities. At this location, the yoga sessions were given within a distance of less than 15 minutes walking. The distance to the workout sessions was about four kilometres from the worksite (a 30-45 minute walk). All guided yoga and workout sessions were given by certified yoga and fitness instructors, respectively. At both locations, the PVC visits were provided near the workplace. Before starting the PVC visits all six coaches attended the same PVC training, at which the aims of the PVC, the items to be discussed as well as the use of the coaching registration form were explained by the principal researcher.

### Implementation of the intervention as planned

#### Dose delivered

**Figure 1:**
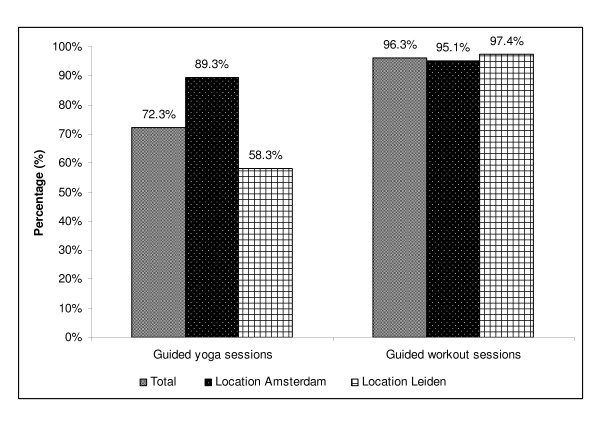
**Dose delivered defined as the percentage of provided yoga and workout group sessions**.

The percentage of provided yoga and workout sessions is illustrated in Figure 1. In total 72.3% of the planned yoga sessions (Amsterdam: 89.3%; Leiden: 58.3%), and 96.3% of all planned workout sessions were indeed provided (Amsterdam: 95.1%; Leiden: 97.4%). As for the provided PVC visits, both locations managed to provide all (100.0%) PVC visits.

#### Fidelity

The intervention protocol with respect to the time schedules of the yoga and workout group sessions was partly followed by the providers. At location Amsterdam, both the yoga and workout sessions were provided on all working days. Each day, there was a yoga session provided during lunchtime, and two or three sessions at the end of the workday. As for the workout sessions, there were every day two or three sessions provided during lunchtime and two sessions at the end of the workday. At location Leiden, the yoga sessions were provided on two working days: one lunchtime session and three sessions were provided at the end of the workday. The workout sessions were provided on four working days: one lunchtime session, one session at the beginning of the workday (i.e. 8 am), and four sessions were provided at the end of the workday. The average size of the provided yoga group sessions was 4.8 workers [min:1, max: 19]. Except for one yoga session, in which 19 workers participated, all other sessions were provided in groups of a maximum of 16 workers. The mean number of workers per guided workout session was 3.9 [min: 1, max: 15]. There were no substantial differences between the two locations regarding the group sizes of the guided yoga.

As to the PVC visits, the mean number of items discussed was 4.3 ± 1.2. There were significant (p < 0.001) more items discussed at location Amsterdam (4.6 ± 1.0) when compared to location Leiden (3.7 ± 1.3). The first two items (goal setting and obtaining confidence in achieving formulated goals) were discussed in 88.8% of all first PVC visits, with no significant differences between locations. The third item, feedback on formulated goals, was discussed in 78.2% of all cases. This was significant (p = 0.011) higher in Amsterdam when compared to Leiden (91.2% versus 79.2%). The fourth and fifth items, discussing barriers for formulated goals and problem solving, were discussed in 64.0% and 65.1% of all cases, respectively. Again, this was significant higher at location Amsterdam (Amsterdam: 91.2% for both items, Leiden: 35.0%: p < 0.001 and 41.0%: p < 0.001, respectively).

### Workers' exposure to the intervention

#### Reach

**Figure 2:**
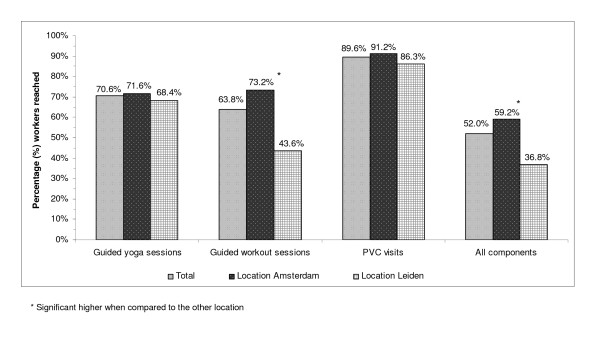
**The percentage of workers that were reached, in total and for locations separate, with regard to the guided group sessions (workout and yoga), PVC visits, and all intervention components together (guided group sessions and PVC visits)**.

The results for the reach of the intervention components are presented in Figure 2.

In total 259 workers (70.6%) of the total intervention group attended at least one yoga session, with no substantial differences between the two locations (71.6% in Amsterdam versus 68.4% in Leiden, χ^2 ^= 0.528). As for the workout sessions, a total of 234 workers (63.8%) of the total intervention group attended at least one guided workout session, with a higher reach among workers in Amsterdam compared to Leiden (73.2% versus 43.6%, χ^2 ^< 0.001). As for the PVC visits, a total of 329 workers (89.6%) attended at least one PVC visit, with no differences between locations (χ^2 ^= 0.153). When taken these three intervention components together, a total of 191 workers (52.0%) attended all three components at least once during the intervention period. This was higher in Amsterdam (59.2%) in comparison with Leiden (36.8%: χ^2 ^< 0.001).

#### Dose received

**Figure 3:**
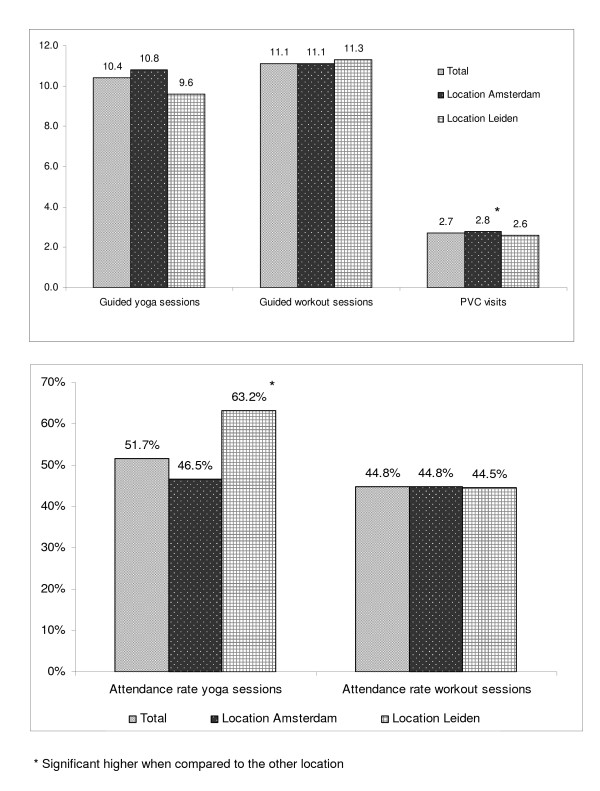
**Dose delivered defined as the mean number of attended guided group sessions and PVC visits, and the attendance rate (%) of the guided yoga and workout group sessions**.

The results for the dose received are presented in Figure 3. The mean number of attended guided yoga sessions was 10.4 (SD = 7.1). The attendance rate of the yoga sessions was 51.7% with a significant higher rate in Leiden when compared to Amsterdam (63.2% versus 46.5%, p = 0.001). Reasons for not attending a guided yoga session were: lack of time, not liking yoga, and health aspects (i.e. musculoskeletal symptoms). For location Leiden, the main reason mentioned for not attending yoga sessions was the time schedule that the yoga sessions were provided. This schedule did not correspond with the regular working hours and only four sessions were offered during the week. As for the guided workout sessions, the mean number of attended sessions was 11.1 (SD = 7.2) and the attendance rate was 44.8%, with no considerable differences between locations (p = 0.938). Reasons for not attending a guided workout session were: lack of time, workers' opinion that they already exercised enough, and not liking to exercise. For location Leiden, the distance to the workout facilities was also mentioned as reason for not attending workout sessions. As for the PVC visits, the mean number of PVC visits per worker was 2.7 (SD = 0.6), which was significant higher (p = 0.001) in Amsterdam when compared to Leiden (2.8 ± 0.5 versus 2.6 ± 0.7). Of all workers in the intervention group, 78.1% (n = 257) attended all three PVC visits, which was significant higher in Amsterdam compared to Leiden (82.9% versus 67.3%, p = 0.005). Reasons for not attending a PVC visit were time constraints and work obligations.

### Workers' attitude towards the intervention

**Figure 4:**
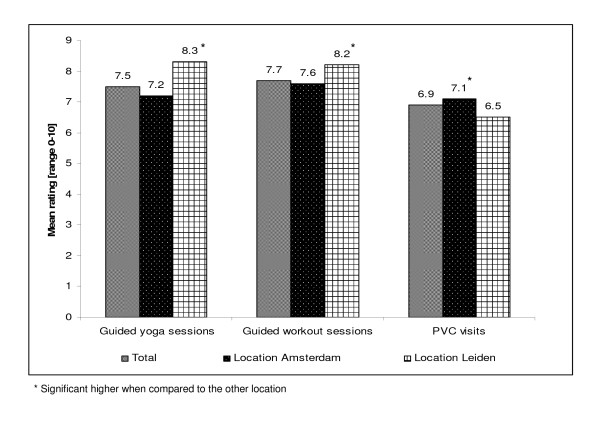
**Workers' opinion with regard to the intervention components**.

By those who attended at least one yoga session (n = 180), a mean score of 7.5 (SD = 1.8) was given (Figure 4), with a significant higher rating in Leiden in comparison with Amsterdam (8.3 ± 1.2 versus 7.2 ± 1.9, p < 0.001). The mean rating of the training guidance of the yoga instructors was 7.7 (SD = 1.6). Again, this was rated significant higher in Leiden when compared to Amsterdam (8.3 ± 1.2 versus 7.4 ± 1.6, p < 0.001). By those having attended at least one workout session (n = 184), an average rating of 7.7 (SD = 1.2) was given, with significant higher rates in Leiden when compared to Amsterdam (Leiden: 8.2 ± 1.0, Amsterdam 7.6 ± 1.3, p = 0.010). The mean rating of the training guidance of the workout instructors was 7.8 (SD = 1.3), with a significant (p = 0.006) higher rating in Leiden (8.3 ± 0.9) than in Amsterdam (7.7 ± 1.3). Those who attended at least one PVC visit (n = 270) rated the PVC visits with a 6.9 (SD = 1.4). The PVC visits were higher rated in Amsterdam when compared to Leiden (Amsterdam: 7.1 ± 1.4, Leiden: 6.5 ± 1.5, p = 0.007).

## Discussion

The study presented in this paper evaluated the process of the Vital@Work intervention using the process relevant elements outlined by Steckler and Linnan [20]: i.e. context, dose delivered, fidelity, reach, dose received, and participants' attitude.

In general, participation levels in worksite health promotion (WHP) programmes have been reported to vary enormously, namely from 10% to 76% [22,23]. In this study, the mean attendance rate of the yoga and workouts sessions was 51.7% and 44.8%, respectively. Regarding yoga, no studies were found that reported attendance rates among working populations. As for the workout sessions, findings of a recent review of Robroek et al. (2009) showed a pooled participation level of 25.8% [range: 22% to 53%] for WHP programmes containing a fitness programme [23]. This pooled participation level was based on six studies. Of these six studies the one of Lechner et al. (1997) was most in line with the Vital@Work study, since the attendance was also registered by the fitness instructors and was on average 53% [24]. Overall, the attendance rate of the guided group session in the Vital@Work study was comparable to rates found in the scientific literature.

The most reported reason for not attending the guided group sessions was a lack of time (both yoga and workout), which has also frequently been reported in the literature as a reason for low physical activity levels [25-27]. Also the study of Kruger et al. (2006) reported that the most commonly mentioned barrier for not using WHP programmes, such as physical activity services (e.g. on-site exercising), were no time during work and lack of time before and after work [28]. A promising solution to overcome the time constraints is to offer employees WHP programmes during paid working time [29]. Although employers may associate this with productivity loss, a good worker health might have the potential to enhance company profitability [30]. This has been suggested since low participation in WHP programmes is associated with lower observed (cost)effectiveness [23,31-33] and even with lower health outcomes, such as higher Body Mass Index (BMI), elevated levels of cholesterol, and higher blood pressure [34]. Although the number of studies investigating determinants of low participation in WHP programmes has increased over the last ten years [28,29,35,36], evidence-based information on how to translate these determinants into appropriate and effective designed methods and strategies to increase participation rates/reach in/of WHP programmes is still lacking.

A possible way to stimulate participation rates in health behaviour research is by tailoring the intervention to specific needs of the target population using the Intervention Mapping (IM) protocol. This six-step protocol for theory- and evidence-based development of health promotion interventions [37,38] was used for the development of the Vital@Work intervention. Based on the focus group interviews held for the needs assessment (step 1 IM), the guided group sessions of the Vital@Work intervention were offered near the workplace, in small group settings, and on times that were most in line with the daily routines of the older workers. This may have resulted in our acceptable attendance rate of the guided yoga and workout sessions, according to the scientific literature indicated earlier. Also, the reach of the guided yoga and workout group session were both satisfactory: 70.6% and 63.8%, respectively. However, the reach of the intervention as a whole (i.e. all intervention components together, Figure 2) was lower than expected: 52.0%. An explanation for this lower reach could be that the chosen strategies to deliver the Vital@Work intervention were based on the determinants of physical activity identified during the focusgroup interviews. However, the intervention itself was aimed at improving two health behaviours, namely vigorous physical activity (i.e. by guided workout sessions) and relaxation (i.e. by guided yoga sessions) [1]. Although we used information obtained from the needs assessment (i.e. step 1 IM) to meet the needs and desires of the older workers, we did not verify whether the combination of the guided yoga and workout group session with the PVC visits indeed appealed to the target population of older workers. A possible explanation for the found differences between the reach of the guided group sessions and PVC separate and the intervention as a whole could be that workers who were interested in yoga were not attracted to involvement in workout sessions and the other way around. Because it is essential to translate the determinants of the intended health behaviour into appropriate strategies that are suitable for the target population [37,39], it is recommended to review the intervention ideas with the intended participants and use their perspectives when choosing the final methods and strategies used to deliver the intervention. Interventions using such an approach appear to be more effective and to have higher participation rates [40].

This study showed some notable differences between the two locations where the Vital@Work intervention was implemented. As for the reach of the workout sessions, this was found to be lower at location Leiden. Several factors may explain the differences observed. First, at both locations the implementation of the Vital@Work intervention was approved by the upper management, which has proven to be essential for the implementation of WHP programmes [19,36,41]. However, at location Leiden there was no written communication toward supervisors and team leaders to document this support. Second, at location Leiden the distance to the workout facilities (about four kilometres) was often mentioned as a reason for not attending a workout session. Workers needed a bicycle or public transport to get there, resulting in a time investment that was considered too much. It is known from research on environmental determinants of physical activity and exercise, that aspects of the physical environment, such as small distance to facilities, positively influence exercise behaviour [42,43]. This was also found to be true for WHP programmes involving physical activity and exercise: easy access to exercise facilities resulted in higher participation rates [35,36,44]. Thus, provision of exercise facilities at the workplace seems promising for improving attendance. The distance to the facilities in Leiden may explain the noteworthy difference concerning the reach of the workout sessions. Although this reach was considerably lower in Leiden, this did not result in lower attendance. This may indicate that the sample in Leiden was a selective group of workers with higher cognitive values towards physical activity and exercise, such as self-efficacy, motivation and health beliefs. For example, workers with a higher motivation are supposed to be more likely to maintain adherence despite large distances or other surveyable barriers. While these cognitive values were not assessed in this study, they have been shown to be an important correlate of adherence to interventions involving physical activity or exercise WHP [45-47]. Another difference between the two locations was the attitude towards both the yoga and workout guided group session, which were rated higher at location Leiden. Two explanations could be addressed for this. First, the earlier mentioned selective sample of motivated workers in Leiden who already appreciated yoga and exercised more than the workers in Amsterdam. Second, during the focus group interviews held to develop the Vital@Work intervention, workers indicated correctly executed training guidance as very important [1]. The training guidance was also rated higher in Leiden, which possibly may have resulted in a higher overall appreciation of the guided group sessions. In contrast to the guided group sessions, the PVC visits were rated lower at location Leiden. This might partly be explained by the fact that, according to the coaching registration forms, the PVCs at this location did not follow the PVC protocol as intended.

## Conclusions

The implementation of the intervention was accomplished as planned with respect to the dose delivered. Most workers were willing to attend the guided group sessions and the PVC visits, although there were differences between the locations and between intervention components. Overall, workers were positive about the intervention.

From this process evaluation, some lessons can be learned for future worksite yoga and physical activity interventions. First, for developers and implementers we recommend making yoga and exercise facilities available near the worksite. Second, a promising solution to overcome the workers' time constraints is to offer employees WHP programme participation during paid working time, and this should therefore be considered by employers. Third, to increase reach of WHP programmes it is necessary to review the eventual intervention ideas with the intended participants and use their perspectives when choosing the final methods and strategies used to deliver the intervention.

## Competing interests

The authors declare that they have no competing interests.

## Authors' contributions

JES, KIP, AvdB, and WvM provided support in the design of the Vital@Work study. JES coordinated the data collection, performed data analysis and drafted the manuscript. KIP, AvdB, and WvM contributed intellectual input and provided support for this study. All authors contributed to the further writing of the manuscript. All authors have read and corrected draft versions of the manuscript and approved the final manuscript.

## Supplementary Material

Additional file 1 **Table 1 - Description of the components of the process evaluation of the Vital@Work intervention**. Click here for file
