# Supplementary material for: A process evaluation of a worksite vitality intervention among ageing hospital workers
Source: Int J Behav Nutr Phys Act. 2011 Jun 10;8:58. doi: 10.1186/1479-5868-8-58 (PMC3127968; doi:10.1186/1479-5868-8-58)
Supplement: Additional file 1 — Table 1 - Description of the components of the process evaluation of the Vital@Work intervention. [file 1479-5868-8-58-S1.PDF]

	Key process component	Purpose	Operationalisation	Measurement
**Context intervention**	Context	Aspects of the environment that may influence the intervention implementation or study outcomes	Description of:<br>1. Organisational factors (i.e. Management support)<br>2. Environmental factors (i.e. Location and facilities of the providers)	NA
**Implementation of the intervention as planned**	Dose delivered	The number of delivered intervention components by the provider	Dose delivered rate (%) of guided yoga and workout group sessions	Attendance registration forms
			Dose delivered rate (%) of the PVC visits	Coaching registration forms
	Fidelity	The extent tot which the intervention was implemented as planned	Attendance providers to time schedules	Attendance registration forms
			Average group sizes guided yoga and workout sessions	Attendance registration forms
			Attendance to PVC protocol: number of items discussed	Coaching registration forms
**Workers' exposure to the intervention**	Reach	The extent to which the workers used the intervention	Number of workers that attended all intervention components at least once	Coaching registration forms<br><br>Attendance registration forms
	Dose received	The extent to which the workers actively were engaged to the intervention	Mean number and attendance rate (%) of guided group sessions	Attendance registration forms
			Mean number of attended PVC visits	Coaching registration forms
**Workers' attitude**	Participants' attitude	Participants' attitude towards the quality of the intervention (i.e. satisfaction)	Workers' opinion per intervention component	Rating on 0-10 scale
			Workers' opinion about the training guidance of the workout and yoga instructors	Scoring on 5-point scale at post-test
